# Deputy Surgeon-General Usher Williamson Evans

**Published:** 1912-09

**Authors:** 


### DEPUTY SURGEON-GENERAL USHER WILLIAMSON EVANS, M.D.

Dr. Evans, late of the Army Medical Department, died in Clifton on August 27th, in his 90th year. He was born in Ireland in 1823, and received his medical education at Dublin, Glasgow and Paris. He entered the army in 1846, and served as a volunteer in the Crimean War, the regiment to which he was attached, the 16th Lancers, being then at home. He was present at the battles of Alma, Balaclava and Inkermann, and did good service in the trenches before Sebastopol, where he helped to organise a transport service of mules to carry the heavy baggage and ammunition of the soldiers to the trenches. He was awarded the medal with four clasps, and the Turkish medal. He became Surgeon-Major in 1866, and retired from the army in 1871 to Westmeath, Ireland. Since 1876 he has lived at Clifton.

Dr. Evans was a keen sportsman, and was a well-known figure for many years as a follower of Lord Fitzhardinge's hounds. He leaves five sons, three of whom are distinguished officers in the army, and two daughters.

